# Beyond the airway: the impact of septoplasty and turbinate reduction on somnolence and sleep disordered breathing risk—a prospective study

**DOI:** 10.3389/fsurg.2026.1828614

**Published:** 2026-06-25

**Authors:** Francisco Alves de Sousa, João Tavares Correia, Marta Rios, Miguel Gonçalves Ferreira, Manuel Magalhães, Mariline Santos

**Affiliations:** 1 Otorhinolaryngology, Head and Neck Surgery, Unidade Local de Saude de Santo Antonio EPE, Porto, Portugal; 2 Pediatrics, Universidade do Porto Instituto de Ciencias Biomedicas Abel Salazar, Porto, Portugal

**Keywords:** daytime somnolence, Epworth sleepiness scale, nasal airflow, peak nasal inspiratory flow, quality of life, septoplasty, sleep-disordered breathing, turbinate reduction

## Abstract

**Introduction:**

Chronic nasal obstruction is a well-recognized driver of daytime somnolence and obstructive sleep-disordered breathing (OSDB). While septoplasty and inferior turbinate reduction (S + ITR) are standard treatments to restore nasal airflow, the relationship between objective gains in Peak Nasal Inspiratory Flow (PNIF) and the resolution of sleepiness remains poorly understood.

**Objective:**

To investigate the impact of S + ITR on daytime somnolence and to determine if these improvements correlate with nasal airflow (PNIF) or anthropometric changes.

**Methods:**

In this prospective study (*N* = 32), patients underwent S + ITR for symptomatic nasal obstruction. Outcomes included the Epworth Sleepiness Scale (ESS), the Berlin Questionnaire (as a screening tool for OSDB risk), airflow (PNIF), and Body composition at baseline and 3 months after surgery.

**Results:**

S + ITR resulted in a significant reduction in daytime somnolence, with ESS scores improving by a mean of 2.7 ± 4.4, *p* = 0.002. Berlin Questionnaire revealed significant improvements in Category 1 (Snoring/Apnea: *p* = 0.024), Category 2 (Somnolence/Fatigue: *p* < 0.001), whereas Category 3 (Systemic/BMI) remained stable (*p* = 0.422). ESS improvement did not correlate with PNIF airflow gains (*p* = 0.516). An inverse correlation was noted between ESS gains and BMI variation (r = - 0.381 for *Δ*ESS vs. *Δ*BMI, *p* = 0.035).

**Conclusion:**

Nasal surgery is associated with a reduction in daytime sleepiness and the screened risk for OSDB. The lack of correlation with PNIF suggests that the surgical benefit for sleepiness may be modulated by other factors beyond nasal airflow improvement. Further research is needed as the exact mechanisms remain to be elucidated.

## 1 Introduction

Nasal breathing (*N*B) is a fundamental physiological process that optimizes inspired air through filtration, humidification, and warming (1, 2). The nasal passages facilitate efficient pulmonary gas exchange via nitric oxide (NO) production and the modulation of neural pathways, such as the trigeminal-vagal reflex (1, 2).

Chronic nasal obstruction, typically caused by septal deviation or turbinate hypertrophy, disrupts these homeostatic mechanisms, forcing a transition to oral breathing (3, 4). This shift is not merely a change in the respiratory route; oral breathing increases airway resistance and instability, which can lead to obstructive sleep-disordered breathing (OSDB) and fragmented sleep architecture (3, 4). Consequently, daytime somnolence may emerge from this nocturnal respiratory inefficiency. Growing evidence suggests that chronic nasal obstruction is linked to OSDB risk and excessive sleepiness, regardless of confounders like age or body mass index (BMI) (5).

While functional nasal surgery—specifically septoplasty and inferior turbinate reduction (S + ITR)—is the gold standard for restoring mechanical patency (6–8), its impact on systemic symptoms like sleepiness is often considered a secondary benefit (9, 10). Current clinical practice relies heavily on Patient-Reported Outcome Measures (PROMs) to evaluate success, yet these tools may not fully capture the recovery of sleep-related alertness (6–8, 11). Furthermore, a significant knowledge gap remains: it is unclear whether the resolution of somnolence results directly from increased airflow volume or from a more complex response to improved respiratory mechanics (9, 10).

The primary aim of this study is to investigate if S + ITR ameliorates subjective daytime somnolence and OSDB risk. Additionally, to test if such improvements correlate with gains in nasal airflow—measured via Peak nasal Inspiratory Flow (PNIF)—or alterations in body composition. By exploring these relationships, we aim to explore how nasal intervention influences daytime alertness and sleep-related health.

## 2 Methods

### 2.1 Cohort selection and surgical protocol

This prospective study evaluated a cohort of patients recruited between January 2024 and October 2024. Eligible participants presented with chronic nasal obstruction attributed to structural abnormalities—specifically septal deviation and inferior turbinate hypertrophy—that had not responded to medical management. To isolate the physiological effects of functional restoration, exclusion criteria were applied: patients with chronic rhinosinusitis, any form of prior nasal surgery, autoimmune diseases, or craniofacial dysmorphisms were excluded.

**Figure 1:**
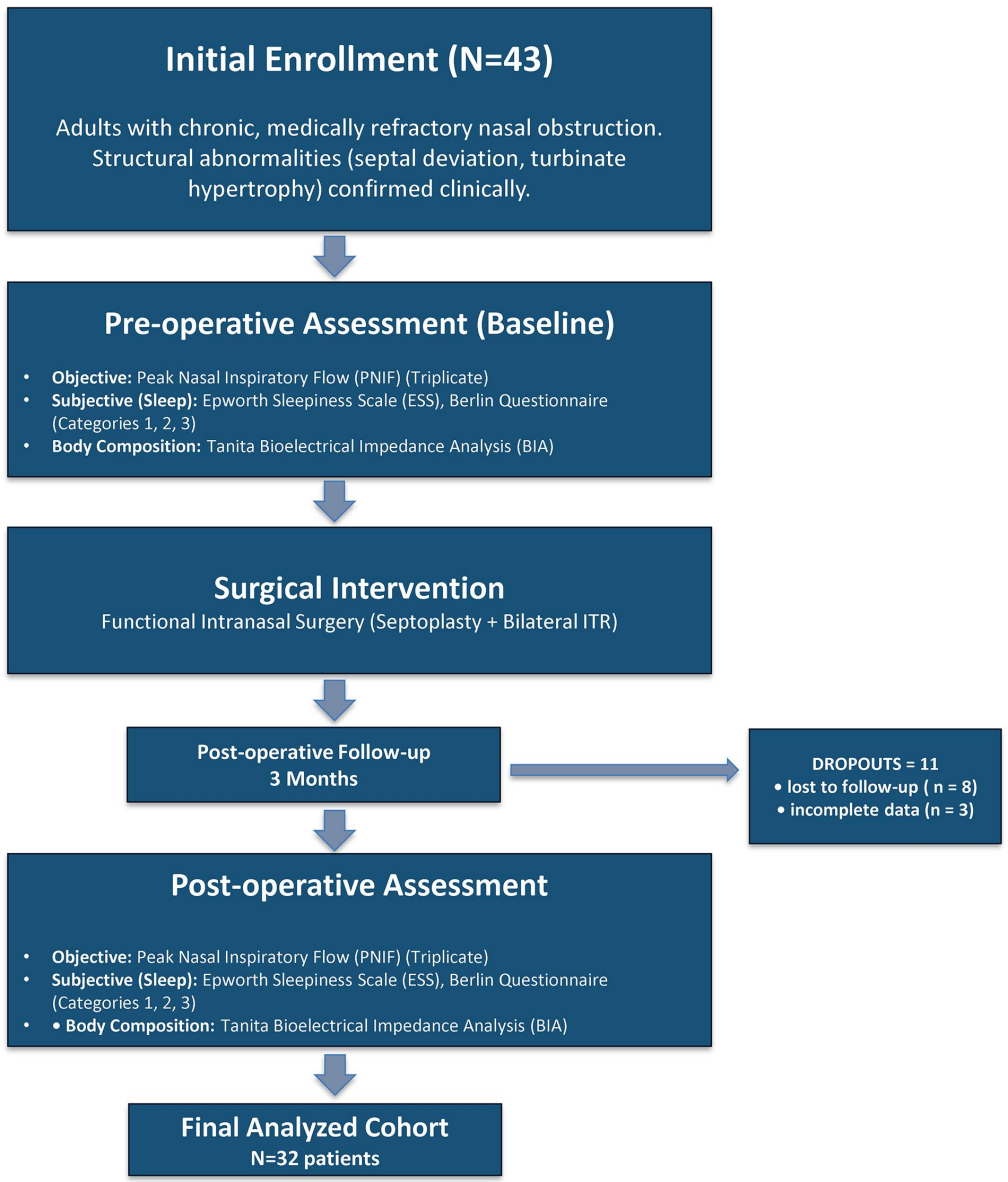
Study Flow and Methodology..

All surgical interventions were performed at a single tertiary center by the same team. The standardized protocol included Cottle technique septoplasty and bilateral inferior turbinate reduction via radiofrequency plasma ablation. Post-operative care followed a uniform regimen, consisting of internal silicone splints for seven days and standardized nasal saline irrigation. The study was conducted in accordance with the Declaration of Helsinki and received formal approval from the local Ethics Committee [ULSSA/ICBAS: 2021–234(186-DEFI/194-CE)]. The methodological workflow is summarized in Figure 1.

### 2.2 Objective patency and anthropometric measurements

Objective nasal airflow was quantified using Peak Nasal Inspiratory Flow (PNIF), a validated and portable metric for assessing nasal patency. Measurements were obtained pre-operatively and at a 3-month follow-up to allow for complete mucosal healing. Patients performed a maximal inspiratory effort through the nose with the mouth closed; the highest value from three consecutive measurements (L/min) was recorded for analysis.

Anthropometric data were obtained via bioelectrical impedance analysis (Tanita UM-076, Tokyo, Japan). The parameters recorded included body weight, muscle mass, body fat percentage, visceral fat rating, and total body water percentage.

### 2.3 Assessment of somnolence and sleep

To assess the impact of surgery on daytime alertness, patients completed the Epworth Sleepiness Scale (ESS), a validated instrument for measuring general daytime sleepiness (12). The ESS is widely used in perioperative and surgical populations (13–15). A change of 2–3 points is generally considered clinically meaningful (16, 17).

The Berlin Questionnaire was employed as a screening tool to categorize patients based on their perceived risk for OSDB. It includes 3 subcategories: snoring/apnea (Category 1), daytime fatigue (Category 2), and obesity/hypertension (Category 3) (18–20). A “High Risk” status is defined as positive scores in at least two categories. Notably, this instrument screens for risk and is not a substitute for polysomnography for a definitive diagnosis of obstructive sleep apnea (OSA). Both ESS and Berlin assessments were conducted at baseline and at the 3-month follow-up.

### 2.4 Statistical analysis

Continuous data are reported as means ± standard deviations. Categorical data are reported as percentages. Differences between pre- and post-operative scores (ESS, PNIF, and BMI) were evaluated using paired t-tests. To investigate the primary research question, Spearman's rho correlated the degree of change in somnolence (*Δ*ESS) with airflow gains (*Δ*PNIF) and body composition alterations (*Δ*BodyWeight, *Δ*BMI, *Δ*MuscleMass). Regarding the Berlin questionnaire, both continuous scores and risk category distributions were compared pre- and post-operatively. The McNemar test was utilized to assess shifts between “High Risk” and “Low Risk” categories. This approach allowed for the analysis of paired nominal data. Finally, repeated measures ANCOVAs evaluated whether the reduction in daytime somnolence (*Δ*ESS) remained significant after adjusting for variations in anthropometric parameters (*Δ*BodyWeight, *Δ*BMI, *Δ*MuscleMass). A separate-model approach was chosen to avoid multicollinearity between interrelated variables like body weight, BMI, and muscle mass. Statistical significance was defined as *p* ≤ 0.05.

## 3 Results

### 3.1 Population demographics

The final analysis included 32 patients who completed the full 3-month follow-up. The cohort consisted of 17 males (53.1%) and 15 females (46.9%), with a mean age of 34.8 ± 12.3 years. Baseline and post operative metrics are summarized in Table 1.

**Table 1:** Outcome variations with surgery (*N* = 32).

Variable	Pre-operative	Post-operative	*P*-value
**Airflow**			
PNIF (L/min)	83.2 ± 30.2	134 ± 44.3	**<0** **.** **001**
**Somnolence/ Sleep**			
ESS Total Score	9.6 ± 5.1	6.8 ± 4.2	**0** **.** **002**
Berlin questionnaire	High risk: 50%	High risk: 25%	0.021^a^
Category 1 - snoring/apnea	1.8 ± 1.7	1.3 ± 1.6	**0** **.** **024**
Category 2 - somnolence/fatigue	1.1 ± 1	0.4 ± 0.8	**<0** **.** **001**
Category 3 - obesity/hypertension	0.2 ± 0.4	0.2 ± 0.4	0.423
**Anthropometrics**			
Total Body Weight (kg)	72.6 ± 16.5	74.4 ± 17.2	**<0** **.** **001**
BMI (kg/m^2^)	24.3 ± 3.6	24.9 ± 3.8	**<0** **.** **001**
Muscle Mass (kg)	51.4 ± 13.2	54.4 ± 13.7	**<0** **.** **001**
Visceral Fat Rating	5.7 ± 3.5	5.3 ± 3.7	0.133
Body Fat (%)	27.3 ± 8.3	24.4 ± 8.5	0.142
Total Body water (%)	50.8 ± 7.3	55.2 ± 9	**0** **.** **040**

Values for continuous variables are expressed as mean ± standard deviation (SD). Categorical variables are expressed as frequencies and percentages (%).

**High Risk Berlin (%):** The proportion of patients classified as high risk for Obstructive Sleep Apnea (positive in ≥ 2 categories).

Statistical Analysis: Paired-samples t-test: Used to compare the pre- and post-operative means for continuous variables.

*P*-value: Significant differences are denoted by *p* ≤ 0.05. Bold values in the table indicate statistical significance.

PNIF, Peak Nasal Inspiratory Flow (L/min); an objective measurement of nasal patency; ESS, Epworth Sleepiness Scale (0–24); a validated tool for assessing daytime somnolence.

Berlin Questionnaire: ^a^Category 1, 2, and 3: Scores representing the severity of snoring/apnea, daytime fatigue, and systemic risk factors, respectively.

*Product of McNemar Test: Used to evaluate the statistical significance of the shift in categorical risk.

### 3.2 Surgical impact

#### 3.2.1 Impact on nasal airflow

Surgical intervention led to highly significant improvements in physical airflow. Mean PNIF values increased from 83.2 ± 30.2 L/min at baseline to 134 ± 44.3 L/min post-operatively (*p* < 0.001 in paired samples t-test).

**Figure 2:**
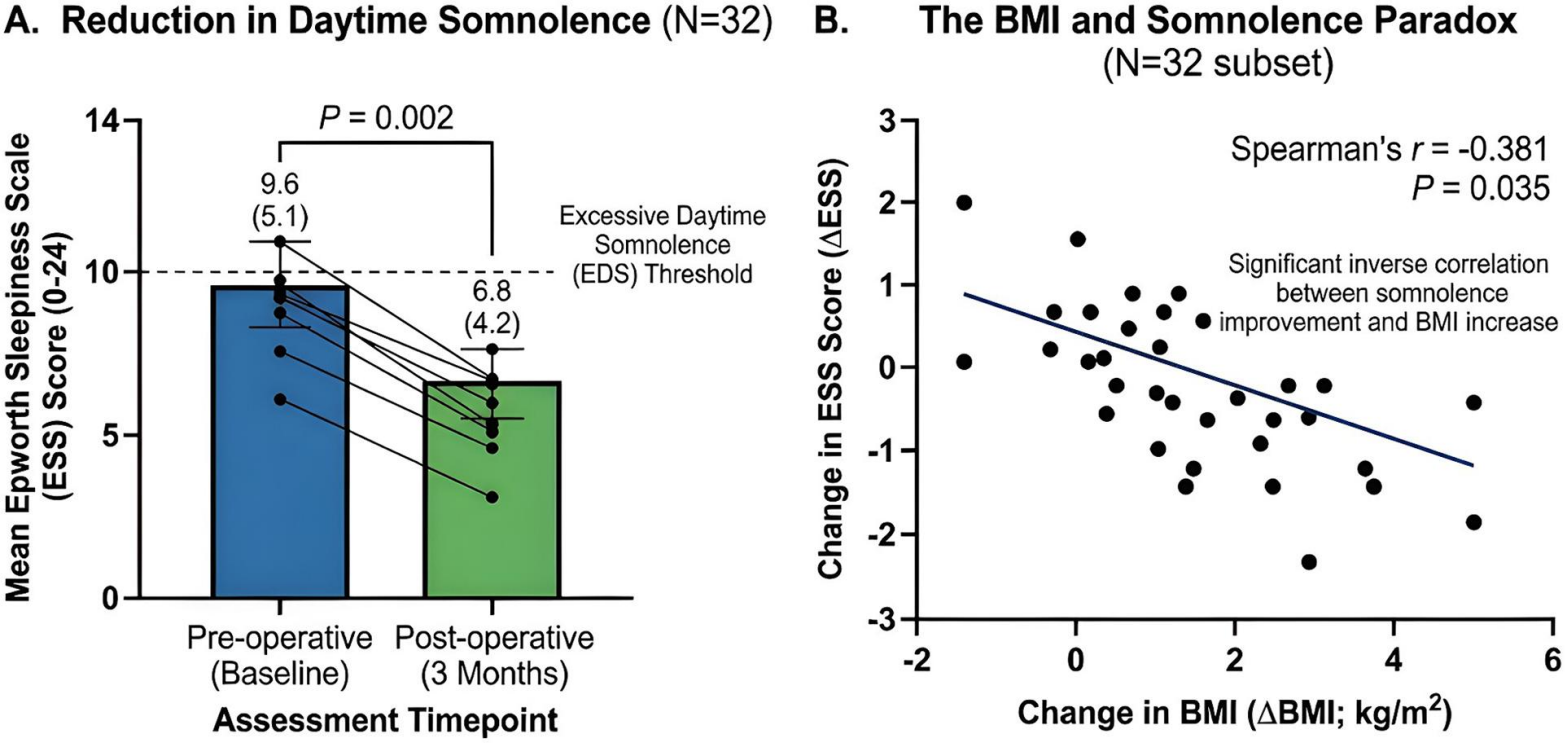
Somnolence outcomes (A) and the Body Mass Index (BMI) paradox (B).

The cohort demonstrated a statistically significant reduction in daytime somnolence, as shown in Figure 2. The mean ESS score improved from 9.6 ± 5.1 to 6.8 ± 4.2 (*p* = 0.002).

Analysis of the **Berlin Questionnaire** revealed significant improvements in the following domains:

- **Category 1 (Snoring/Apnea):** Decreased from 1.8 ± 1.7 to 1.3 ± 1.6 (*p* = 0.024).
- **Category 2 (Somnolence/Fatigue):** Decreased from 1.1 ± 1 to 0.4 ± 0.8 (*p* < 0.001).
- **Category 3 (Systemic Factors/BMI):** Remained stable (0.2 ± 0.4 vs. 0.2 ± 0.4, *p* = 0.423).

There was a significant reduction in the proportion of patients classified as “**High Risk” for OSA**. Pre-operatively, **50% (*N*** **=** **16)** of the cohort were high-risk, dropping to **25% (*N*** **=** **8)** following surgery (*p* = 0.021, McNemar test). Notably, eight patients transitioned from high to low risk, while none moved from low to high risk.

Significant post-operative variations were observed in body weight, BMI, and muscle mass. Detailed mean changes are presented in Table 1.

### 3.3 The airflow-somnolence correlation

As shown in Table 2, no significant correlation was found between the magnitude of airflow gain (*Δ*PNIF) and the reduction in daytime sleepiness (*p* = 0.516). As displayed in Figure 2 a significant inverse correlation was observed between changes in sleepiness and changes in BMI (r = −0.381, *p* = 0.035).

**Table 2:** Bivariate correlation analysis (Pearson's correlation) between post-pre operative variations (*N* = 32).

Variable 1 (*Δ*)	Variable 2 (*Δ*)	Correlation Coefficient (r)	*P*-value
PNIF Variation	ESS Variation	0.097	0.516
Weight Variation		−0.330	0.070
BMI Variation		−0.381	0.035*
Muscle Mass Variation		−0.214	0.266
Total Body Water Variation		−0.085	0.661

* Statistically significant (*p* < 0.05). The negative correlation between *Δ*BMI and *Δ*ESS indicates that as BMI slightly increased, daytime somnolence significantly decreased.

Repeated measures ANCOVAs were performed to determine if the reduction in somnolence was independent of body composition changes:

#### 3.3.1 Controlling for weight

The main effect of time was not statistically significant, F (1, 29) = 1.84, *p* = 0.186, *η*^2^ = 0.060, indicating that somnolence scores did not differ significantly between the pre-test and post-test when accounting for the covariate. The interaction between time and weight approached, but did not reach, statistical significance, F(1, 29) = 3.55, *p* = .070, *η*^2^= 0.109.

#### 3.3.2 Controlling for BMI

The main effect of time was not statistically significant, F(1, 29) = 1.66, *p* = 0.208, *η*^2^ = 0.054, indicating that somnolence scores did not differ significantly between the pre-test and post-test when accounting for the covariate. The interaction between time and BMI variation was not statistically significant, F(1, 29) = 3.25, *p* = 0.082, *η*^2^= 0.101, though it continues to show a slight trend toward significance, similarly to the weight model.

#### 3.3.3 Controlling for muscle mass

The main effect of time was statistically significant, F(1, 27) = 5.14, *p* = 0.032, *η*^2^ = 0.160, indicating that somnolence scores decreased significantly between the pre-test even when accounting for the covariate. The interaction between time and muscle mass variation was not statistically significant, F(1, 27) = 1.29, *p* = 0.266, *η*^2^ = 0.046.

## 4 Discussion

### 4.1 The impact of nasal surgery on sleepiness

The results indicate that S + ITR significantly alleviates daytime somnolence, as evidenced by a mean improvement of 2.7 points in the Epworth Sleepiness Scale (ESS). This aligns well with published literature: meta-analyses show ESS improvements ranging from −1.5 to −4.7 points after nasal surgery (13, 21–23). While the exact underlying cause remains unknown, we hypothesize that restoring a functional nasal airway might reduce the respiratory effort required during sleep, potentially decreasing micro-arousals and stabilizing sleep architecture (22, 24).

Snoring and fatigue categories of the Berlin Questionnaire also showed significant improvement, as documented in Literature (25–27). It is important to reiterate that these results reflect a reduction in the clinical risk profile rather than a confirmed resolution of sleep apnea, as objective sleep studies were not performed.

### 4.2 The dissociation between airflow and alertness

A key finding is the absence of a statistically significant correlation between objective airflow gains (*Δ*PNIF) and the reduction in somnolence (*p* = 0.516). This lack of association suggests that the improvement in sleepiness with S + ITR might not depend solely on raw nasal inspiratory volume gains. While our study did not measure specific airflow dynamics, qualitative factors—such as improved laminar flow or a reduction in the work of breathing after surgery—may play a role.

Literature suggests that nasal breathing may influence autonomic pathways, such as the trigeminal-vagal reflex (28–31). Although we cannot confirm these mechanisms with our current data, it is possible that improved nasal patency supports more restorative sleep through enhanced sensory feedback or altered parasympathetic tone, independent of absolute volume increases.

A significant inverse correlation was observed between the change in ESS and the change in BMI (r = −0.381, *p* = 0.035), indicating that patients who gained more weight actually experienced the greatest improvements in alertness. While weight gain is typically associated with increased sleepiness, our data suggest that these changes were primarily driven by increases in muscle mass (Table 1). This might indicate a recovery of lean mass once sleep-disordered breathing is mitigated, though this interpretation remains speculative.

We hypothesize that restoring nasal breathing could relate to increments in muscle mass trough mitigation of a chronic, respiratory-induced catabolic state (28–36). The potential involvement of metabolic or hormonal axes—such as growth hormone or the ghrelin-leptin system—also merits future investigation (37–41). However, as our study did not include objective metabolic biomarkers, these observations should be treated as purely hypothetical. Further research is required to determine if nasal breathing influences systemic recovery and energy homeostasis beyond its role as a respiratory conduit (42).

Several limitations warrant consideration. Due to the exploratory nature of the study, a formal pre-study power calculation was not performed. The modest sample size (*N* = 32) and single-arm design without a control group limit the generalizability of these findings and the ability to exclude external lifestyle influences. Additionally, the reliance on the subjective Berlin Questionnaire rather than objective polysomnography prevents a definitive confirmation of sleep apnea resolution or changes in the Apnea-Hypopnea Index. The study is further constrained by a short three-month follow-up and the use of bioelectrical impedance analysis, which is less precise than dual-energy x-ray absorptiometry for assessing body composition shifts.

## 5 Conclusion

Functional intranasal surgery is associated with decreased daytime somnolence and a lower clinical risk profile for sleep apnea. These improvements appear to be independent of the absolute magnitude of airflow gains and may be significantly modulated by the patient's anthropometric profile. However, as these findings are based on subjective screening tools and patient-reported outcomes, they should be interpreted with caution. Future research utilizing objective sleep studies and metabolic biomarkers is necessary to confirm these systemic benefits and their long-term sustainability.

## Data Availability

The datasets presented in this article are not readily available because the Local Ethics Committee approval limits database sharing with third party. Requests to access the datasets should be directed to the corresponding author.
